# The Occurrence of Multidrug Resistant Bacteria in the Urine of Healthy Dogs and Dogs with Cystitis

**DOI:** 10.3390/ani9121087

**Published:** 2019-12-05

**Authors:** Andreia R. Yamanaka, Alessandra T. Hayakawa, Ícaro S. M. Rocha, Valéria Dutra, Valeria R. F. Souza, José N. Cruz, Lázaro M. Camargo, Luciano Nakazato

**Affiliations:** 1 Faculty of Veterinary Medicine, University of Cuiabá, Cuiabá, MT 78065-700, Brazil; andreia.vet@hotmail.com (A.R.Y.); lazaro.camargo@kroton.com.br (L.M.C.); 2 Faculty of Veterinary Medicine, Federal University of Mato Grosso, Cuiabá, MT 78060-900, Brazil; tammyhito@gmail.com (A.T.H.); icarosmrocha@hotmail.com (Í.S.M.R.); valeriadutra.dutra@gmail.com (V.D.); valeriaregia27@gmail.com (V.R.F.S.); 3 Department of Statistics, Federal University of Mato Grosso, Cuiabá, MT 78060-900, Brazil; niltonn.cruz@gmail.com

**Keywords:** dogs, urine, microorganisms, urinary tract infection, multidrug resistance, carrier

## Abstract

**Simple Summary:**

Antimicrobial resistance is a global health issue. The “One Health” concept describes animals and environments playing an important role in the resistance to antimicrobials. In this study, we survey healthy companion animals (dogs) as a potential source of multidrug resistant (MDR) bacteria, and compare them with dogs with cystitis. Both groups have a similar isolated genus profile and frequency of multidrug resistance. In our study, both healthy and cystitis animals were found to be carriers of MDR bacteria.

**Abstract:**

The objectives of this study were to evaluate the occurrence of microorganisms, the antimicrobial susceptibility profile, and the presence of multidrug resistant (MDR) bacteria in the urine of clinically healthy dogs and dogs with cystitis. The urine was collected through cystocentesis. Subsequently, culture and antimicrobial susceptibility tests were performed. The isolates were classified based on their resistance profile, to evaluate the presence of MDR bacteria. Statistical analyses were performed using the chi-squared or Fisher’s exact tests. Bacterial isolates were present in 24.39% of the dogs in the control group, and 60.27% of the dogs in the cystitis group. The cystitis group was associated with a higher risk of bacterial isolates than the control group (odds ratio = 7.5; 95% confidence interval = 2.81–22.40). The main isolates were *Staphylococcus* spp., *Escherichia coli*, *Proteus* spp., and *Enterobacter* spp. in both groups. A high percentage of isolates were resistant to ampicillin in both groups. The lowest resistance presented by the isolates in both groups was to meropenem. Only the resistance to quinolones was different between the groups. The proportions of MDR isolates were 70% (7/10) and 65.91% (29/44) in the control and cystitis groups, respectively. The results showed the presence of MDR bacteria in the urine of both the healthy dogs, and the dogs with cystitis. The drug resistance was high, reinforcing the importance of establishing an effective treatment approach against urinary tract infections in pets, to minimize the spread of bacterial resistance and its impact on public health.

## 1. Introduction

Bacterial urinary tract infections (UTIs) are a common cause of morbidity in dogs. They occur in approximately 14% of dogs throughout their lives, more commonly in neutered female and older dogs, at an average age of 7–8 years [1,2,3]. Several bacteria have been isolated, such as *Escherichia coli, Staphylococcus* spp., *Proteus* spp., *Klebsiella* spp., *Enterococcus* spp., and *Streptococcus* spp. [2,4,5,6,7].

These bacteria are commonly found in the urine and may be associated with the multidrug resistant (MDR) phenotype to antibiotics [3]. However, healthy dogs might have bacteriuria and positive urine culture tests [8], but data on the profile of antimicrobial resistance are scarce.

There have only been a few studies in Brazil on antimicrobial resistance in dog urine isolates. Therefore, in this study, we aimed to evaluate the profile of microorganisms, patterns of antimicrobial susceptibility, and the presence of MDR bacteria in the urine of clinically healthy dogs and dogs with UTIs.

## 2. Materials and Methods 

### 2.1. Animals

The animals were divided into two groups—the control group, including 41 clinically healthy dogs without clinical signs of UTIs or other conditions, and without concomitant or recent treatment (three months) with antimicrobials and urinalysis, and the cystitis group, including 73 dogs clinically diagnosed with cystitis based on the clinical signs of polyuria, dysuria, stranguria, hematuria, or a combination of these signs, and without concomitant or recent treatment with antimicrobials. The study was approved by the Animal Use Ethics Committee of the Federal University of Mato Grosso (23108.184285/2016-03).

### 2.2. Urine Collection 

All collections took place from February to December 2016 in the Imaging and Medical Clinic of the Small Animals sector of the Veterinary Hospital of the University of Cuiabá and the Federal University of Mato Grosso. The urine was collected using the cystocentesis technique via a 25 × 7 mm hypodermic needle and a 10 mL syringe under ultrasound guidance [2,5]. The healthy dogs were examined to detect the presence of urolithis. We collected 2 mL of urine using an aseptic technique. The urine samples were stored in sterile vials and processed within 1 h of collection.

### 2.3. Bacterial Culture and Antimicrobial Susceptibility Testing

From the urine samples stored in microtubes, an aliquot (1 mL) was seeded on 8% Sheep Blood agar, MacConkey agar, and Sabouraud agar plus non-chloramphenicol, and incubated at 30 °C and 37 °C for 5 days. Morphological and biochemical identifications were performed based on the report by Quinn et al. [9].

The antibiogram technique with the disk diffusion method was performed on the isolates [10], and nine classes of antibiotics [11] were tested: β-lactams (penicillin, cephalosporins, and carbapenems), aminoglycosides, phenicol, quinolones, tetracyclines, nitrofurans, and sulfonamides. The antibiotics tested were ampicillin (AMP), amoxicillin, amoxicillin + clavulanic acid, amikacin, cephalexin, ceftiofur, enrofloxacin (ENO), ciprofloxacin (CIP), imipenem, meropenem, chloramphenicol, doxycycline, and nitrofurantoin. Isolates resistant to three or more antimicrobial categories were classified as MDR [12].

### 2.4. Statistical Analysis

A logistic regression analysis was performed for the qualitative variables, such as the gender, race, age, group (control/cystitis), and isolation of the MDR bacteria (yes/no). Statistical significance was considered as *p*-values ≤ 0.20 [13]. The chi-squared or Fisher’s exact test was used to compare the profile of the bacterial genera, the percentage of bacterial resistance, and the percentage of MDR bacteria between the control and cystitis groups [14].

## 3. Results

### 3.1. Clinical Data of Dogs

In the control and cystitis groups, 24.39% (10/41) and 60.27% (44/73) of the animals, respectively, had bacterial isolates. Thus, the cystitis group was associated with a higher risk of bacterial isolates compared to the control group (odds ratio [OR] = 7.5; 95% confidence interval [CI] = 2.81–22.40). The age of the animals varied from 3 months to 15 years ($\bar{X}$ = 5.69 and S = 3.83) in the cystitis group, and from 4 to 19 years ($\bar{X}$ = 9.83 and S = 4.18) in the control group. Table 1 shows the descriptions of the main variables.

### 3.2. Microbiological Analysis of Urine

**Figure 1:**
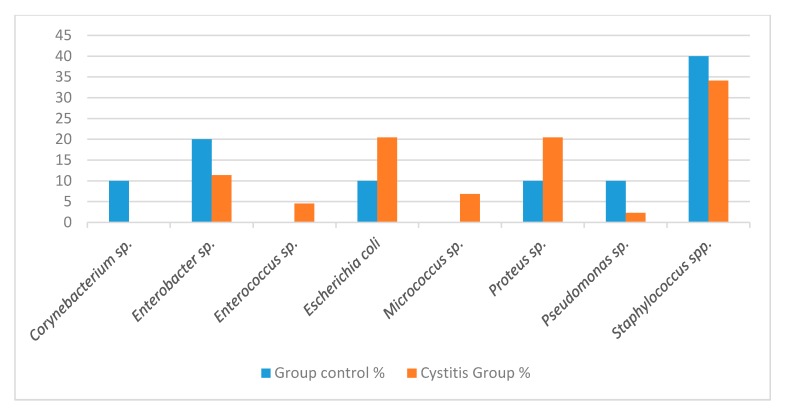
The percentage of bacterial isolates from the urine of dogs in the control (n = 10) and cystitis groups (n = 44).

Profiles of the isolated bacteria species are depicted in Figure 1. The main isolates were of *Staphylococcus* spp., *Escherichia coli*, *Proteus* spp., and *Enterobacter* spp., with different isolation percentages among the groups, but without statistical significance.

### 3.3. Antimicrobial Analysis of Isolates

Table 2 shows the antimicrobial susceptibility pattern of both groups. A high percentage of isolates were resistant to AMP, ENO, and marbofloxacin (MBF) in the cystitis group, and to AMP, nitrofurantoin, and chloramphenicol in the control group. Comparing the susceptibility pattern between the groups, only the quinolone class (ENO, CIP, and MBF) had a statistical difference (*p* < 0.05). The lowest resistance in both groups was to meropenem.

The proportions of MDR isolates were 70% (7/10) and 65.91% (29/44) in the control and cystitis groups, respectively. However, animals in the cystitis group had a higher chance of presenting with an MDR bacterium (OR = 4.3; 95% CI = 1.57–1.61). Supplementary Table S1 shows the distribution of urinary MDR bacterial isolates in the cystitis and control groups.

## 4. Discussion

In this study, a lower percentage (24.39%) of urinary bacterial isolates was observed in the control group, but the bacterial and antimicrobial resistance profiles, including the percentage of MDR bacteria, were similar in both groups.

MDR bacteria have increased in recent years and are a big public health challenge, causing therapeutic limitations in humans and animals. The concept of “One Health” was introduced in relation to MDR bacteria, recognizing that human, animal and environmental health are interconnected and affect one another (Interagency Coordination Group on Antimicrobial Resistance) [15].

Several studies have shown that urine is not sterile. Studies on the human urinary microbiome have identified the presence of microorganisms even in healthy populations [16,17,18,19,20,21]. It has recently been found that the canine urinary bladder is not a sterile environment [20]. There were fewer bacterial isolates in the control group than in the cystitis group, similar to the study on humans by Tang [22].

In this study, *Staphylococcus* spp. was the most isolated bacterium in both groups. However, in the cystitis group, there was a proportional, although not statistically significant, increase in fecal isolates of *Escherichia coli*, *Enterococcus* spp., and *Proteus* spp. Wong et al. [23] reported *Escherichia coli* and *Staphylococcus* spp. as the most prevalent bacteria in that order. The highest occurrence of *Staphylococcus* spp. in both groups may be associated with the cystocentesis technique of urine collection [4].

The antibiotic resistance profiles were similar in both groups. However, the resistance to quinolones (MBF, ENO, and CIP) was higher in the cystitis group. This fact may be associated with the increase in *Escherichia coli* isolates in the cystitis group compared to the control group, with resistance in this species being common [24]. Marques, et al. [6] reported an increase in the resistance of *Staphylococcus* spp. to fluoroquinolones.

The microbial susceptibility pattern showed a low percentage of resistance to meropenem, imipenem, and amikacin in the tested isolates in both groups. The use of carbapenems should be restricted in UTIs. Carbapenems should be administered only in cases of MDR UTIs. Their restricted use is probably a factor influencing their high antimicrobial sensitivity [3,25].

Other antibiotics with a low resistance in the cystitis group were amoxicillin + clavulanic acid, with 25% resistance, and ceftiofur, with 29% resistance. The low resistance of ceftiofur reported by Ferreira, et al. (2014) was 26% [26]. The new diagnostic and treatment guidelines for UTIs show a high prevalence of resistance to amoxicillin, and a low prevalence of resistance to clavulanic acid [3], similar to our study showing a 54% resistance to amoxicillin and a 25% resistance to amoxicillin + clavulanic acid.

The occurrence of MDR bacteria in the urine was similar in both groups. MDR bacteria are common in the respiratory, oral, fecal, and skin microbiota of healthy humans [27]. Based on the occurrence of MDR bacteria in healthy dogs, any antibiotic treatment, if necessary, must always assess the urine culture to establish the criteria for the choice of antibiotics [28], to improve patient clinical signs and avoid MDR spread. The increase in MDR bacteria in the urine leads to limited therapeutic options for veterinary use. Moreover, the need to prescribe antimicrobials for human use is increasing [6,29].

According to Sorensen et al. [7], the over-prescription of antibiotics is common in dogs with suspected UTIs, and the use is inappropriate and unnecessary in most cases. Culture and antimicrobial susceptibility testing should be performed to choose the appropriate antibiotic. Veterinarians should be made aware of the generation of MDR traits with use of antimicrobials in animals [24].

## 5. Conclusions

The results of this study showed a similar occurrence of bacterial profile, antibiotic resistance, and percentage of MDR bacteria in both groups, reinforcing that the urine of healthy dogs also has colonization of MDR bacteria. This suggests the need for antimicrobial susceptibility testing in dogs with UTIs since, in addition to establishing an effective therapeutic approach, we seek to minimize the occurrence of MDR bacteria and preserve the future usefulness of available antimicrobial agents.

## Figures and Tables

**Table 1:** Variable clinical data of control and cystitis groups of dog urine samples.

Variables		Control Group (n = 41)		Cystitis Group (n = 73)	
		n	%	n	%
Sex	Female	31	75.61	39	53.42
	Male	10	24.39	34	46.58
Race	Mixed	15	36.58	24	32.88
	Pincher	4	9.76	7	9.59
	Shitzu	3	7.32	6	8.22
	Others	19	46.34	36	49.31

**Table 2:** Antimicrobial susceptibility profile in urinary isolates from dogs in the control and cystitis groups.

Antimicrobials Classes	Agent	Resistance (%)	
		Cystitis (n = 44)	Control (n = 10)
Penicillins: β-lactams	AMO	54.55% (24)	30% (3)
	AMC	25% (11)	20% (2)
	AMP	68.18% (30)	50% (5)
Cephalosporins: β-lactams	CFE	50% (22)	30% (3)
	CTF	29.55% (13)	30% (3)
Carbapenems: β-lactams	MPM	10% (4)	0% (0)
	IPM	15.91% (7)	20% (2)
Aminoglycosides	AMI	13.64% (6)	10% (1)
Quinolones	ENO ^a^	61.36% (27)	10% (1)
	CIP ^a^	50% (22)	10% (1)
	MBF ^a^	59.09% (26)	10% (1)
Phenicol	CLO	38.64% (17)	40% (4)
Tetracyclics	DOX	50% (22)	10% (1)
Nitrofurans	NIT	38.63% (17)	50% (5)
Sulfonamides	SUL	56.82% (25)	30% (3)

AMO: amoxicillin, AMC: amoxicillin + clavulanic acid, AMP: ampicillin, CFE; cephalexin, CTF: ceftiofur, MPM: meropenem, MPI: imipenem, AMI: amikacin, ENO: enrofloxacin, CIP: ciprofloxacin, MBF: marbofloxacin, CLO: chloramphenicol, DOX: doxycycline, NIT: nitrofrimurin, Sot: nitrofrimantoin. ^a^ significant statistic between groups (*p* < 0.05).

## Supplementary Materials

The following are available online at https://www.mdpi.com/2076-2615/9/12/1087/s1, Table S1, Frequency distribution of MDR bacteria genera in urine of dogs (Healthy and Cistitis).
