# Supplementary material for: The Occurrence of Multidrug Resistant Bacteria in the Urine of Healthy Dogs and Dogs with Cystitis
Source: Animals (Basel). 2019 Dec 5;9(12):1087. doi: 10.3390/ani9121087 (PMC6941014; doi:10.3390/ani9121087)
Supplement: Supplementary file 1 [file animals-09-01087-s001.pdf]

Suplementary material

Table S1. Frequency distribution of MDR bacteria genera in urine of dogs (Healthy and Cistitis)

Genera	Control (n= 7)	Cistitis (n= 29)	Total (n= 36)
Proteus sp.	14.28% (1/7)	31.03% (9/29)	27.77% (10/36)
Staphylococcus sp.	14.28% (1/7)	20.69% (6/29)	19.45% (7/36)
Escherichia coli	14.28% (1/7)	17.24% (5/29)	16.67% (6/36)
Enterobacter sp.	28.60% (2/7)	17.24% (5/29)	19.45% (7/36)
Pseudomonas	14.28% (1/7)	3.45% (1/29)	5.55% (2/36)
Micrococcus	0% (0/7)	3.45% (1/29)	2.78% (1/36)
Enterococcus	0% (0/7)	6.90% (2/29)	5.55% (2/36)
Corynebacterium sp	14.28%(1/7)	0 (0/29)	2.78% (1/36)
